# A quick review of infrared thermography studies on children and adolescents’ mental health

**DOI:** 10.3389/fpsyt.2025.1515032

**Published:** 2025-09-19

**Authors:** Jing Zhao, Xu Hong, Xinran Zhang, Ying Li, Yiming Ma, Ziying Zhang, Siyu Huo, Yudi Sun

**Affiliations:** ^1^ College of Art and Design, Beijing University of Technology, Beijing, China; ^2^ Beijing Children’s Hospital, Capital Medical University, National Center for Children Healthy, Beijing, China

**Keywords:** infrared thermography, mental health, emotional disorders, ASD, PTSD, stress

## Abstract

**Introduction:**

Infrared Thermography (IRT) is valuable for monitoring surface temperature distributions, with proven benefits in assessing physiological states. However, most research focuses on adults, neglecting its potential for evaluating children and adolescents, whose mental health significantly impacts learning and social adaptation. This study aims to review the applications of IRT in different psychological health fields and identify gaps in its clinical diagnostic standards for pediatric psychological assessment.

**Methods:**

A rapid review approach is employed to search for literature on the applications of IRT in emotional disorders, Autistic Spectrum Disorder (ASD), Post traumatic stress disorder (PTSD), stress, and cognitive load from PubMed, ScienceDirect, Google Scholar and PLOS ONE. The included articles were subjected to manual screening and qualitative analysis. After screening 2395 citations and excluding low-quality ones, 27 out of 50 reviewed articles were included.

**Results:**

For children, IRT can capture facial temperature changes caused by autonomic nervous system responses to emotional disorders. In ASD, IRT can detect temperature changes related to emotional and cognitive states, enhancing diagnosis and insights into sensory processing and emotional regulation. Additionally, IRT can capture the emotional, stress, and psychological responses of PTSD patients. For cognitive load, relying solely on self-reporting frequently lacks objectivity, while IRT offers a non-invasive, real-time method across various scenarios.

**Discussion:**

IRT has revealed correlations between physiological reactions and changes in body temperature detectable by thermal imaging, leading to methods integrating IRT with biometric measurement techniques and creating datasets for assessing individual conditions. This advancement supports evaluating psychological health in children and adolescents.

## 1 Introduction

Infrared Thermography (IRT) is a powerful non-invasive technology used to measure the temperature distribution of object surfaces. IRT generates thermal images by detecting the infrared radiation emitted by objects, providing detailed information about surface temperatures. This technology functions effectively under various lighting conditions, allowing for stable data acquisition even in environments with significant variability. Since it does not need direct contact with the object being measured, IRT is particularly suitable for collecting physiological data without disturbing the subjects (1).

Studies have shown that IRT can capture the temperature distribution characteristics of the human body, and changes in mental health status can be reflected in body temperature variations. IRT technology can objectively record the temperature characteristics under changes in mental health status, and then infer a person’s mental health status. For example, changes in facial temperature are one of the commonly used values to observe changes in a person’s mental state. When a person is anxious, the forehead heat distribution may be abnormally high; while in a depressed state, the facial metabolic heat may be abnormally low; when a person is tired, depressed, etc., the temperature of the eye area tends to rise; when a person is happy or joyful, the temperature around the eye socket and the chin area will rise. Therefore, IRT technology has been increasingly applied in the field of mental health. With the maturity of IRT technology in the field of mental health, it has shown unprecedented potential in mental health fields such as emotional disorders, autism spectrum disorder (ASD), post-traumatic stress disorder (PTSD), stress and cognitive load, providing researchers with an intuitive perspective to capture the mental health dynamics that are difficult to reach with traditional methods, and providing valuable scientific basis for subsequent psychological treatment.

At present, the application of IRT in the field of mental health is more concentrated in the study of adult mental health, and there are relatively few studies on the mental health of children and adolescents. Due to its non-invasive nature, real time meansturement capability and device portability, IRT shows great potnetial for application in adolescents mental health assessments. Given the limitations of children and adolescents in accurately expressing their subjective psychological feelings, traditional data collection 49 methods often face challenges. IRT technology cleverly bypasses the obstacles of children and adolescents in accurately expressing their subjective psychological feelings, and provides an unprecedented innovative solution for the mental health assessment of this age group. It can intuitively and instantly capture the changes in the surface temperature of the children and adolescents being tested. As an objective indicator of mental health status, it effectively makes up for the problem of insufficient subjective description and opens up a new path for the study of mental health of children and adolescents.

Therefore, in the context of children and adolescents’ mental health research, the application of IRT technology has paved the way for a more comprehensive and in-depth understanding of children’s mental wellbeing. This article aims to lay a solid theoretical foundation for subsequent researchers in this field to explore the specific application strategies of IRT in the field of mental health, its actual effects, and foresee its future development trends through a detailed literature review and comprehensive analysis. The study systematically reviewed the effectiveness of IRT in the assessment of mental health, such as emotional disorders, ASD, PTSD, stress, and cognitive load. By critically analyzing existing research results, it was used to reveal how IRT, with its non-invasive and real-time characteristics, makes up for the limitations of traditional assessment methods and achieves the goal of improving the accuracy and efficiency of mental health assessment. Through this study, it is hoped that the means of mental health assessment can be enriched, and a contribution can be made to improving the level of mental health services and promoting the healthy growth of children and adolescents.

## 2 Methods

### 2.1 Search strategy and selection of studies

This study conducted a systematic review of the literature on “IRT in Children and Adolescent Mental Health Research.” The purpose was to investigate the impact of thermal infrared imaging technology on the psychological health of children and adolescents. A comprehensive search strategy was employed, covering all English-language articles published in conferences and journals from 2000 to 2024. The application of IRT in the field of psychology has become increasingly sophisticated since 2000, improving the understanding of its ability to capture psychological states, with a corresponding increase in research in this area (2, 3). However, excluding early foundational research may limit exploration of the historical development and early applications of IRT. Although these foundational studies are of significant value, in order to ensure that the review reflects the key trends in technological advancements and clinical applications, this study limits its scope to the period between 2000 and 2024.

We conducted a comprehensive search using the PubMed, ScienceDirect, Google Scholar, and PLOS ONE databases. In articles within the medical field, we used “Infrared thermography” or “Thermal imaging” as the primary keywords for the search. Furthermore, we used keywords such as “Emotional disorders”, “Anxiety”, “Depression”, “ASD”, “PTSD”, “Stress responses”, and “Cognitive load” to conduct a categorical search for relevant studies in which IRT was used as the primary assessment method.

We applied inclusion and exclusion criteria to ensure the relevance and quality of the selected studies. The inclusion criteria were as follows: (1) published between 2000 and 2024; (2) written in English; (3) focused on the application of IRT in psychophysiological research; and (4) explored mental health issues such as emotional disorders (e.g. anxiety, depression), ASD, PTSD, stress, or cognitive load. The exclusion criteria were as follows: (1) published outside the specified date range; (2) not in English; (3) not using IRT as the primary assessment method or detection tool; (4) not exploring mental health or psychophysiological processes; and (5) being duplicate articles, irrelevant, or focusing on irrelevant topics.

**Figure 1:**
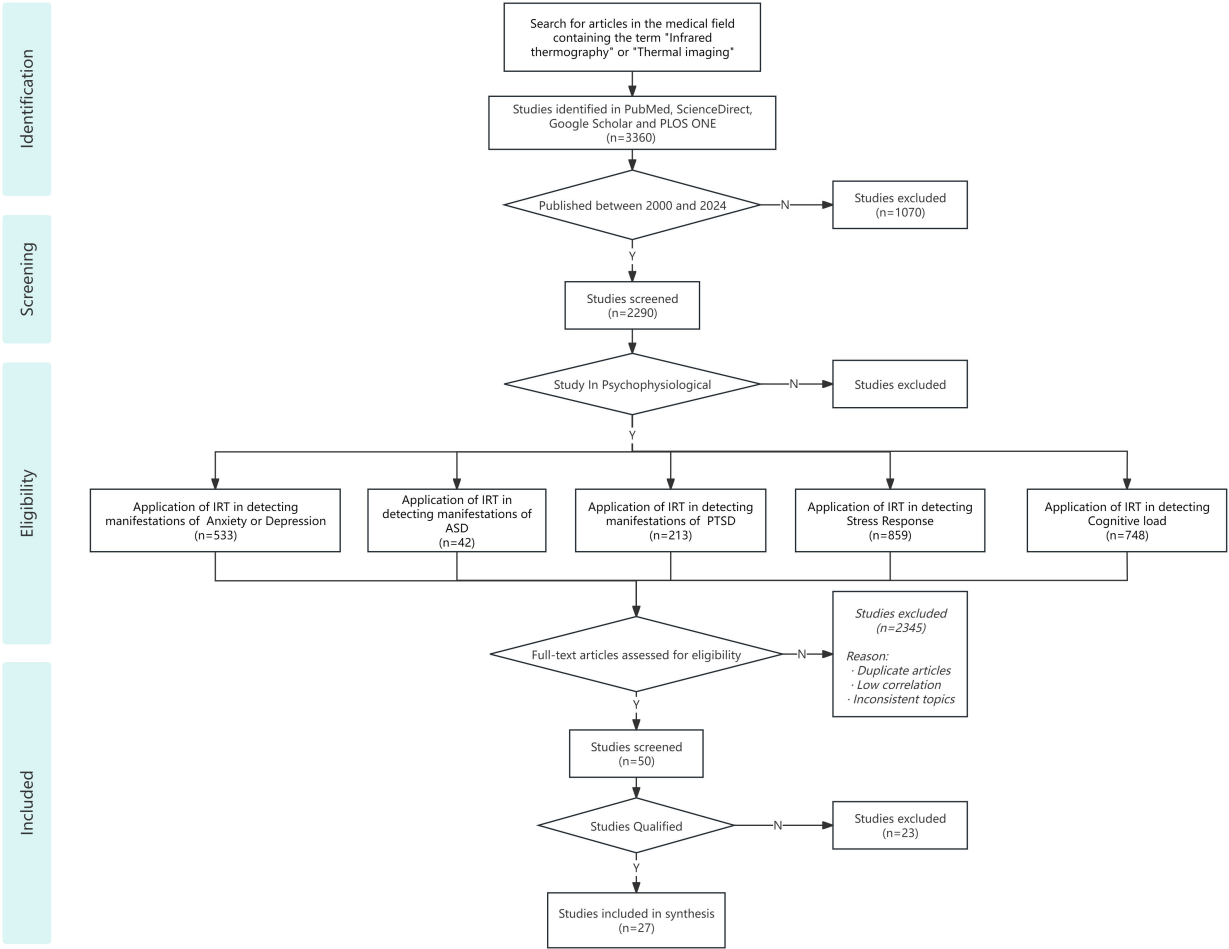
The flowchart for the literature search and screening process.

A preliminary search yielded a total of 3360 results. After excluding articles published outside the specified time frame, 2290 articles were selected based on their titles and abstracts. Further screening was conducted to identify studies related to these mental disorders that utilized IRT as an assessment tool. Following eligibility assessment and the exclusion of duplicates, studies with low relevance, and unrelated topics, a final set of 50 studies was retained. Following a manual review of the literature for qualification, the articles suitable for inclusion in the study were identified. The flowchart for the literature search and screening process based on data is illustrated in the **Figure 1** below.

### 2.2 Quality assessment

To enhance the selection process and ensure high-quality articles, we utilized the Quality Assessment Tool for Studies with Diverse Designs (QATSDD) to assess the quality of the studies included. Initially created for psychological research. Originally developed for psychological research, the QATSDD tool has been increasingly applied across a broad range of health service research, extending to fields such as auxiliary medical care, medicine, public health, nursing, health services, and social sciences, due to its robust reliability (4). The QATSDD has demonstrated strong reliability and validity (Kappa = 71.5%) in quality assessment across various studies (5).

The QATSDD tool consists of 16 criteria, including “Explicit theoretical framework,” “Statement of aims/objectives in main report,” and “Clear description of research setting.” Each criterion is rated on a scale from 0 (no information) to 3 (detailed information), where 0 indicates the absence of information or poor quality, 1 indicates minimal or vague information, 2 indicates adequate or moderate quality, and 3 indicates comprehensive and high-quality information. For example, Criterion 1: Explicit Theoretical Framework, a score of 3 indicates a clearly defined and well-established theoretical framework that directly influences the study design, while a score of 0 indicates no mention of a theoretical framework. The total possible score for each article is 48 points. Studies with a total score of 36 or higher are considered to have high methodological quality; studies with scores between 24 and 35 are considered to have acceptable quality, while studies with scores below 24 are regarded as having significant deficiencies, which may affect the credibility of the research findings. To ensure consistency and objectivity in the evaluation process, scoring was performed independently by two reviewers. Any disagreement in scoring was resolved through discussion or third-party review. Ultimately, 27 of 50 articles initially identified are selected and prioritized for inclusion in the study.

### 2.3 Data extraction

We extract the following data based on each included study: (1) author; (2) year; (3) research contents; (4) types of mental illness; (5) sample size; (6) camera used; (7) measurement tools, including the psychological 122 assessment tools, data analysis tools, machine learning methods, biosignal measurement methods, and 123 follow-up; and (8) main findings.

## 3 Results

### 3.1 Characteristics of included studies

Using the QuADS tool to filter out articles that do not meet the criteria from an initial pool of 50 articles, 27 relevant studies are identified. Of these, 7 articles focus on emotional disorders, 6 on Autism Spectrum Disorder (ASD), 3 on Post-Traumatic Stress Disorder (PTSD), 5 on stress-related topics, and 6 on cognitive load. Among the 27 included studies, 9 specifically target pediatric populations (i.e. children and adolescents up to 18 years of age), with many of the remaining studies including mixed age samples or not specifying the age of the participants.

The 27 studies incorporate two types of non-contact infrared devices: Infrared Thermal Cameras and Infrared Thermal Imager, both employing IRT technology. Specifically, 7 studies use Infrared Thermal Cameras, 18 use Infrared Thermography, and 2 studies utilize both devices. In terms of measurement tools, aside from using psychological assessment tools such as GAD-7 and Cooper-Harper, 7 studies combine IRT with various physiological measurement methods, including Galvanic Skin Response (GSR), Respiratory Variability Spectrum (RVS), and Electroencephalogram (EEG). Additionally, 5 studies employ data processing and analysis tools like OpenCV Library, GraphPad Prism, and MATLAB to develop neural network models for identifying and classifying mental health issues. Among the main findings of these studies, all 27 indicate a relationship between body heat distribution and emotional states or mental health disorders. Specifically, 15 studies highlight the role of facial temperature, suggesting that monitoring changes in facial heat distribution can serve as a diagnostic indicator. Furthermore, 6 studies propose system models based on IRT technology for rapidly identifying physiological responses across different psychological states, addressing limitations of traditional methods and expanding application scenarios. Collectively, these studies provide new insights into the application of IRT technology in assessing emotional and physiological states, contributing to enhanced perspectives on mental health evaluation and the diagnosis of mental disorders. **Table 1** presents literature on IRT and mental health research involving children and adolescents. **Table 2** summarizes the characteristics and methodological features of the 27 studies included in this review, categorized by research focus, participant demographics, device types, and key findings related to the application of IRT in mental health assessment.

**Table 1:** A review of research on IRT and adolescent mental health.

Researchers [Ref.]	Year	Study	Types of mental illness	N	Camera used	Measurement	Main findings
Septiadi et al. (6)	2019	Differences in anxiety conditions at facial temperatures measured using thermal imaging	Anxiety	40	FLIR SC5000	General Anxiety Disorder	The results showed that more increased the temporal and frontal of face temperature, more severe the anxiety. There is a significant negative relationship between face temperature and anxiety level (*p* < 0.05).
San Fernández et al. (7)	2023	Physiological indices of social anxiety	Anxiety	/	Infrared thermal imaging camera	Social Anxiety Questionnaire for Adults	The temperature of the tip of the nose decreased significantly in participants with low (vs. high) social anxiety (*p* < 0.001), while no significant differences were found in other facial regions of interest: forehead (*p* = 0.999) and cheeks (*p* = 0.999).
I. Pavlidis et al. (8)	2001	A distance facial patterns of anxiety, alertness, and/or fearfulness	Anxiety	6	An uncooled thermal camera by Raytheon	Image analysis methods	In anxious states blood is locally redirected to the periorbital musculature to facilitate rapid eye movements.
Gasparro et al. (9)	2021	Evaluating if dental anxiety can be measured objectively using thermal infrared imaging	Anxiety	60	ENV-THIMG-M, Zaventem, Belgium	Modified Dental Anxiety Scale (MDAS)	Correlation between the thermal imaging measurements and the scores of the MDAS questionnaire was found for nose and ear, but not for all of the other regions.
Sulistiowati et al. (10)	2017	Determining differences in temperature distribution in the face of students who experience anxiety	Anxiety	81	Infrared thermal imaging camera	General Anxiety Disorder, Spearman test correlation	Mild until severe anxiet y level happens when the temperature is increasing.
Goulart et al. (11)	2019	Emotion analysis in children through facial emissivity of infrared thermal imaging	Depression	28	Therm-App infrared thermal imaging camera	SAM (Self-Assessment Manikins)	The emissivity variations were an efficient marker to analyze emotions in facial thermal images, and IRTI was confirmed to be an outstanding technique to study emotions.
Maller et al. (12)	2016	Major depressive disorder (MDD) and corneal temperature	Depression	32	NEC F30S Thermo Shot	Hamilton Depression Rating Scale (HAMD)	Corneal temperature may be an indicator of clinical severity in psychiatric disorders, including depression.
Haputhanthri, D et al. (13)	2020	Integration of Facial Thermography in EEG-based Classification of ASD	ASD	32	ICI, Inc.	Cross-validation with EEG data	A computational model named ASDGenus is proposed, indicating that thermal imaging features can be used together with EEG as potential biomarkers for autism classification.
Ganesh K et al. (14)	2021	Deep learning techniques for automated detection of autism spectrum disorder based on thermal imaging	ASD	100	Infrared thermal imaging camera	Customized convolutional neural network and ResNet 50 network	The temperature difference of anger was the largest in the eye, cheek and nose regions of autistic and non-autistic participants.
M. Melinda et al. (15)	2023	A Novel Autism Spectrum Disorder Children Dataset Based on Thermal Imaging	ASD	34	FLIR E95	A deep learning model based on convolutional neural network (CNN) classifies the faces of autistic children based on thermal imaging.	Using CNN Tensorflow for training and validation. The proposed dataset of autistic children based on thermal imaging can be applied to the identification of autistic children.
N. Rusli et al. (16)	2020	Implementation of Wavelet Analysis on Thermal Images for Affective States Recognition of Children With Autism Spectrum Disorder	ASD	57	T420	Develop an emotional state classifier for children with ASD	An emotional state model classifier for autistic children based on thermal imaging is proposed.
Muthiah et al. (17)	2024	Hotspot Detection Pada Citra Termal Wajah Anak Autis Dan Normal Berbasis Otsu Thresholding	ASD	34	Infrared thermal imaging camera	Otsu threshold is used for hot spot detection	The face classification model of autistic children using thermal images was developed using the general CNN architecture.
Coben, R et al. (18)	2008	Infrared Imaging and Neurofeedback: Initial Reliability and Validity. Journal of Neurotherapy	ASD	37	ThermoVision A20M camera (FLIR Systems, 2006)	Cross-validation with EEG data	Changes in thermal regulation during and between treatment courses of autism spectrum disorders are related to the reduction of core symptoms and the improvement of brain connectivity.
Cardone D et al. (19)	2015	Thermal Infrared Imaging Based Computational Psychophysiology for Psychometrics	PTSD	8	Infrared thermal imaging camera	GSR and facial thermal IR imaging analysis	Thermal infrared imaging can be used to examine fear conditioning in post-traumatic stress disorder, and the analysis of facial thermal responses during the conditioning paradigm can detect disease-related sympathetic responses.
Cruz-Albarran et al. (1)	2016	Human emotions detection based on a smart-thermal system of thermographic images	PTSD	25	Infrared thermal imaging camera	Thermal image analysis methods	The intelligent thermal imaging system can monitor biomedical thermal images, detect ROI, and diagnose emotions by automatically analyzing biomarkers of temperature.
Cardone D et al. (20)	2017	New Frontiers for Applications of Thermal Infrared Imaging Devices: Computational Psychopshysiology in the Neurosciences	PTSD	59	Infrared thermal imaging camera	GSR and facial thermal IR imaging analysis	Thermal infrared imaging combined with standard GSR can be used to study fear processing in PTSD.
Engert V et al. (21)	2014	Exploring the use of thermal infrared imaging in human stress research	Stress	15	High resolution thermal infrared (IR-) imaging	Cold Stress Test (CPT) and Trier Social Stress Test (TSST)	Using high-resolution infrared thermal imaging technology to obtain sympathetic nerve activity indicators, this method is better than the traditional method, and can be used to collect data related to special populations.
Pavlidis I et al. (22)	2012	Fast by nature-how stress patterns define human experience and performance in dexterous tasks	Stress	17	Mid-wave infrared (MWIR) camera from FLIR (model SC6000)	State-Trait Anxiety Inventory (SAI) and Thermal image analysis methods	The instantaneous sweating response of the perinasal area is measured by thermal imaging to quantify the pressure. This measurement method is real-time and has less impact on user activities, and has a wider application scenario.
Ioannou S et al. (23)	2013	The autonomic signature of guilt in children: a thermal infrared imaging study	Stress	20	Digital FLIR SC3000 thermal infrared camera	ThermalCAM Researcher by FLIR	Under the pressure of guilt, children’s peripheral nasal vessels contracted, nasal tip temperature decreased, and body temperature decreased.
Cho Y et al. (24)	2017	Deep learning of breathing patterns for automatic stress recognition using low-cost thermal imaging in unconstrained settings	Stress	8	Thermal imager	Respiration variability spectrogram (RVS) and Deep Convolutional Networks (CNNs)	By detecting the change of temperature around the nostril a deep learning model that can automatically identify the level of psychological stress according to people’s breathing patterns is proposed.
Cho Y et al. (25)	2019	Instant stress: detection of perceived mental stress through smartphone photoplethysmography and thermal imaging	Stress	17	Smartphone and thermal camera	Automatic feature learning of artificial neural networks	They proposed a system that combines PPG and thermography with the aim of improving cardiovascular signal quality and detecting stress responses quickly.
Abdelrahman, Y et al. (26)	2017	Exploring the Usage of Thermal Imaging to Unobtrusively Estimate Cognitive Load	Cognitive Load	12	Optris PI1607 and PI45010	OpenCV library9 for image processing and facial points extraction; The Stroop test with four levels of difficulty	Using computer vision to analyse forehead and nose temperature differences in thermal camera footage to estimate four levels of cognitive load, we found that the metric has a strong correlation with task difficulty in both artificial control and natural tasks, suggesting its potential for real-time cognitive load assessment
Or et al. (27)	2007	Development of a Facial Skin Temperature-based Methodology for Non-intrusive Mental Workload Measurement	Cognitive Load	33	Thelmal imaging camera MikroScan noov (Mihon)	A non-invasive facial skin temperature measurement method; The revised Cooper-Harper psychometric test	Using infrared thermography to display changes in cognitive workload through the revised Cooper-Harper test;the same facial skin temperature measuring method can be used in a field and in a laboratory setting.
Elizabeth M et al. (28)	2021	Physiological indicators of task demand, fatigue, and cognition in future digital manufacturing environments; International Journal of Human-Computer Studies	Cognitive Load	36	Artinis Octamon and fNIRS device, Zephyr Bioharness 3, FLIR thermal imager	Physiological measurement data; subjective data; background experience; demographic survey; NASA-TLX questionnaire; the 9-point fatigue rating scale; Matlab	Fatigue has a small but significant effect on heart rate, breathing rate, and hemodynamic responses. Task demand significantly affects breathing rate and nasal temperature, but no differences in heart rate or hemodynamic responses across task demand levels were observed.
Wang R et al. (29)	2023	Evaluation of facial temperature distribution changes during meditation using infrared thermal imaging: An experimental, cross-over study	Cognitive Load	32	Medical IRTI system	GraphPad Prism software; Mindful Attention Awareness Scale (MAAS); Big Five Inventory (BFI)	An increase in chin temperature may be a representative feature of a meditation state, and forehead temperature is also a potential indicator.
Panasiti et al. (30)	2019	Cognitive load and emotional processing in psoriasis: a thermal imaging study	Cognitive Load	33	FLIR^©^ camera SC3000	The Emotional N-Back Task; Functional Infrared Thermal Imaging (fITI) technology	Patients with psoriasis may experience less emotional demand under high cognitive load conditions. Additionally, patients benefit more from the load-dependent interference effect when processing emotional information.
Kang et al. (31)	2006	Determining learning level and effective training times	Cognitive Load	9	MikronScan 7200V Thermal Camera	A non-invasive facial skin temperature measurement method; The revised Cooper-Harper psychometric test	Nose temperature and accuracy increased when learners’ reaction time and subjective load perception decreased.

**Table 2:** Summary of the included studies on IRT and mental health.

Aspects of included studies	Category	Number of studies	Number of studies on pediatric
Types of Mental Illness	Emotional disorders	7	1
	ASD	6	6
	PTSD	3	/
	Stress	5	2
	Cognitive load	6	/
Participant Demographics	Children and adolescents	9	/
	Mixed-age or unspecified	18	/
IRT Device Type	Infrared thermal cameras Infrared thermography	7 18	6 3
	Both	2	/
	Combining IRT with physiological sensors	7	1
Measurement	Data processing and analysis Tools	5	9
Key Findings	Association between thermal patterns and psychological states Facial temperature as diagnostic indicator	27 15	9 2
	Development of mental health assessment system model based on IRT	6	2

Among the 9 articles focusing on children and adolescents, the ages of the participants range from 3 to 18 years old. There are 6 articles targeting ASD, while only 2 and 1 studies focus on anxiety and depression respectively. There are 5 studies that employ emotion-induction tasks such as audiovisual stimulation or interactive play, with task durations ranging from 10 to 20 minutes. In terms of thermal data analysis, 4 studies select facial regions including the periorbital area, nasal tip, forehead, and cheeks as regions of interest (ROIs), with 2 studies specifically highlighting significant temperature changes at the nasal tip in the experimental group. Furthermore, 5 studies apply machine learning methods, such as Convolutional Neural Networks (CNN), linear discriminant analysis (LDA), Bayes and Random Forest, to classify emotional or diagnostic states, achieving accuracy rates of up to 98%. Although the number of studies specifically targeting children and adolescents remains limited, existing research has demonstrated the initial feasibility and potential of this technique in pediatric psychological assessment. Moreover, the extensive application of IRT in adult populations provides methodological references and empirical support for its adaptation in younger cohorts. While this review does not exclusively focus on pediatric samples, the inclusion of studies across age groups offers valuable insights into the broader applicability of IRT for emotional state recognition in children. At the same time, it underscores the pressing need for future research to further explore this population and expand beyond ASD to encompass a wider range of psychological conditions.

### 3.2 Quality assessment

After an overall assessment of the quality of the literature by reviewers using the QATSDD tool, it is observed that most articles exhibit a high quality level. See **Table 3** for content. Many studies feature clear theoretical frameworks, well-defined objectives, and comprehensive research backgrounds, with mean scores above 2.5 for Criteria 1 to 3. This suggests that experiments using IRT in various psychological and psychiatric issues possess solid research significance and systematic experimental design, contributing to the rigor and scientific nature of quantitative research.

**Table 3:** Quality assessment of the included studies based on the QATSDD tool.

The included studies		1	2	3	4	5	6	7	8	9	10	11	12	13	14	15	16	Total score
1	Septiadi et al.	3	3	2	1	3	3	3	2	2	3	N/A	3	3	N/A	N/A	2	33
2	Fernández et al.	3	3	3	2	1	2	2	1	2	2	N/A	2	2	N/A	0	2	27
3	I. Pavlidis et al.	2	3	1	0	1	3	3	1	2	3	N/A	2	2	N/A	0	1	24
4	Gasparro et al.	3	3	3	2	2	2	2	2	1	3	N/A	3	3	N/A	N/A	2	31
5	Sulistiowati et al.	2	2	2	1	3	1	1	0	2	3	N/A	2	3	N/A	N/A	1	23
6	Goulart et al.	3	3	3	2	2	2	1	1	2	3	N/A	3	3	N/A	0	3	31
7	Maller et al.	3	1	3	2	2	2	1	2	2	3	N/A	3	3	N/A	0	3	30
8	Haputhanthri, D et al.	3	3	3	3	2	1	2	2	3	3	N/A	2	2	N/A	N/A	3	32
9	Ganesh K et al.	2	3	3	2	2	3	2	2	2	2	N/A	2	2	N/A	N/A	1	28
10	M. Melinda et al.	2	3	2	3	2	2	2	2	3	2	N/A	3	2	N/A	1	2	31
11	N. Rusli et al.	2	2	2	1	1	3	2	3	2	3	1	3	2	0	N/A	1	28
12	Muthiah et al.	2	1	2	2	2	3	1	2	2	3	N/A	3	1	N/A	0	2	26
13	Coben, R et al.	2	3	3	3	2	3	2	3	3	2	N/A	2	3	N/A	N/A	1	32
14	Or et al.	3	3	3	1	2	3	2	3	1	3	N/A	3	2	N/A	N/A	3	32
15	Abdelrahman, Y et al.	2	3	3	1	1	2	3	1	2	3	N/A	3	2	N/A	N/A	3	29
16	Elizabeth M et al.	3	2	3	2	3	2	3	1	3	3	N/A	3	2	2	N/A	1	33
17	Wang R et al.	2	2	2	2	3	3	2	2	1	3	N/A	3	2	N/A	N/A	1	28
18	Panasiti et al.	3	3	3	0	2	1	3	2	1	2	N/A	2	1	1	N/A	2	26
19	Kang, J et al.	3	3	2	1	1	2	2	0	2	3	N/A	2	2	N/A	N/A	0	23
20	Cardone D et al.	3	2	2	1	2	1	2	1	1	2	N/A	3	2	N/A	1	2	25
21	Cruz-Albarran I A et al.	3	3	3	2	3	1	2	3	2	3	N/A	1	2	1	1	2	32
22	Cardone D et al.	3	2	3	2	2	2	3	2	3	3	N/A	2	2	1	0	3	33
23	Engert V et al.	3	3	2	2	2	2	3	1	2	2	N/A	2	2	N/A	0	2	28
24	Pavlidis I et al.	3	3	3	2	2	3	3	2	1	2	N/A	2	2	N/A	0	2	32
25	Ioannou S et al.	3	3	3	2	2	1	1	2	1	2	N/A	2	3	N/A	0	1	26
26	Cho Y et al.	2	3	2	3	1	3	2	3	2	1	N/A	3	2	N/A	0	2	29
27	Cho Y et al.	2	3	3	3	3	2	2	2	1	2	N/A	1	1	N/A	0	3	28
Criteria																		
1: Explicit theoretical framework 2: Statement of aims/objectives in main report 3: Clear description of research setting 4: Evidence of sample size considered in terms of analysis 5: Representative sample of target group of a reasonable size 6: Description of procedure for data collection 7: Rationale for choice of data collection tool(s) 8: Detailed recruitment data 9: Statistical assessment of reliability and validity of measurement tool(s) (Quantitative studies only) 10: Fit between research question and method of data collection (Quantitative studies only) 11: Fit between research question and format and content of data collection tool e.g. interview schedule (Qualitative studies only) 12: Fit between research question and method of analysis (Quantitative studies only) 13: Good justification for analytic method selected 14: Assessment of reliability of analytic process (Qualitative studies only) 15: Evidence of user involvement in design 16: Strengths and limitations critically discussed																		

However, there are shortcomings in terms of sample size considerations, recruitment data, and statistical evaluation of measurement tools. The mean score for evidence on sample size and detailed recruitment data is 1.78, suggesting that the sample size may be insufficient or not representative. Additionally, the mean score for the statistical evaluation of the reliability and validity of measurement tools is below 2, which may compromise the credibility of the research findings. Standard 14, for which most articles are deemed not applicable, indicating that the experimental design may not be consistent with the actual situation of users or the effect of simulating emotions such as stress and cognitive load in the experiment is generally moderate. Overall, while the quality of the articles is commendable, there is room for improvement. Addressing these issues could significantly enhance the reliability and validity of the experiments, ensuring that research results are more accurate and practically meaningful.

### 3.3 The principle of IRT and applications in the field of mental health

IRT is a technology that detects and records changes in the surface temperature of objects using infrared radiation. The human body exhibits subtle temperature variations based on different physiological states and emotional responses, which IRT can monitor and analyze. In the mental health field, IRT offers a non-invasive, real-time, and objective method for assessing an individual’s psychological state and emotional responses (32, 33). This technology provides researchers with key physiological information about mental health status by monitoring thermal distribution across various body parts.

IRT could capture the temperature distribution characteristics of the human body under different psychological conditions. Generally, mental health states manifest through changes in temperature distribution across several areas, including the face, chest, abdomen, limbs, and back. Facial temperature variations are the most prominent and easily detectable, especially around the nose tip, eye region, and forehead (34). These areas’ temperature changes are often associated with emotional fluctuations and stress responses. For instance, studies show that in states of anxiety or tension, the temperature of the nose tip may decrease, while the temperature around the forehead and eyes may increase (34). The temperature distribution in the chest and abdominal regions is also closely linked to psychological states. During intense emotional reactions, such as fear or anger, the chest area may exhibit a tendency for increased temperature due to enhanced blood flow from sympathetic nervous system activation. Temperature changes in the limbs are relatively minor; however, in extreme psychological states, the temperature of the palms and feet may decrease due to peripheral vasoconstriction (35). The temperature changes in the back often correlate with overall psychological and physiological states, such as increased temperature in the shoulder blade area due to higher stress or tension.

By analyzing temperature changes across different body regions, particularly the face, researchers better understand an individual’s physiological responses under various psychological conditions. This analysis can provide important physiological indicators for mental health assessments.

#### 3.3.1 IRT equipment and data collection

**Figure 2:**
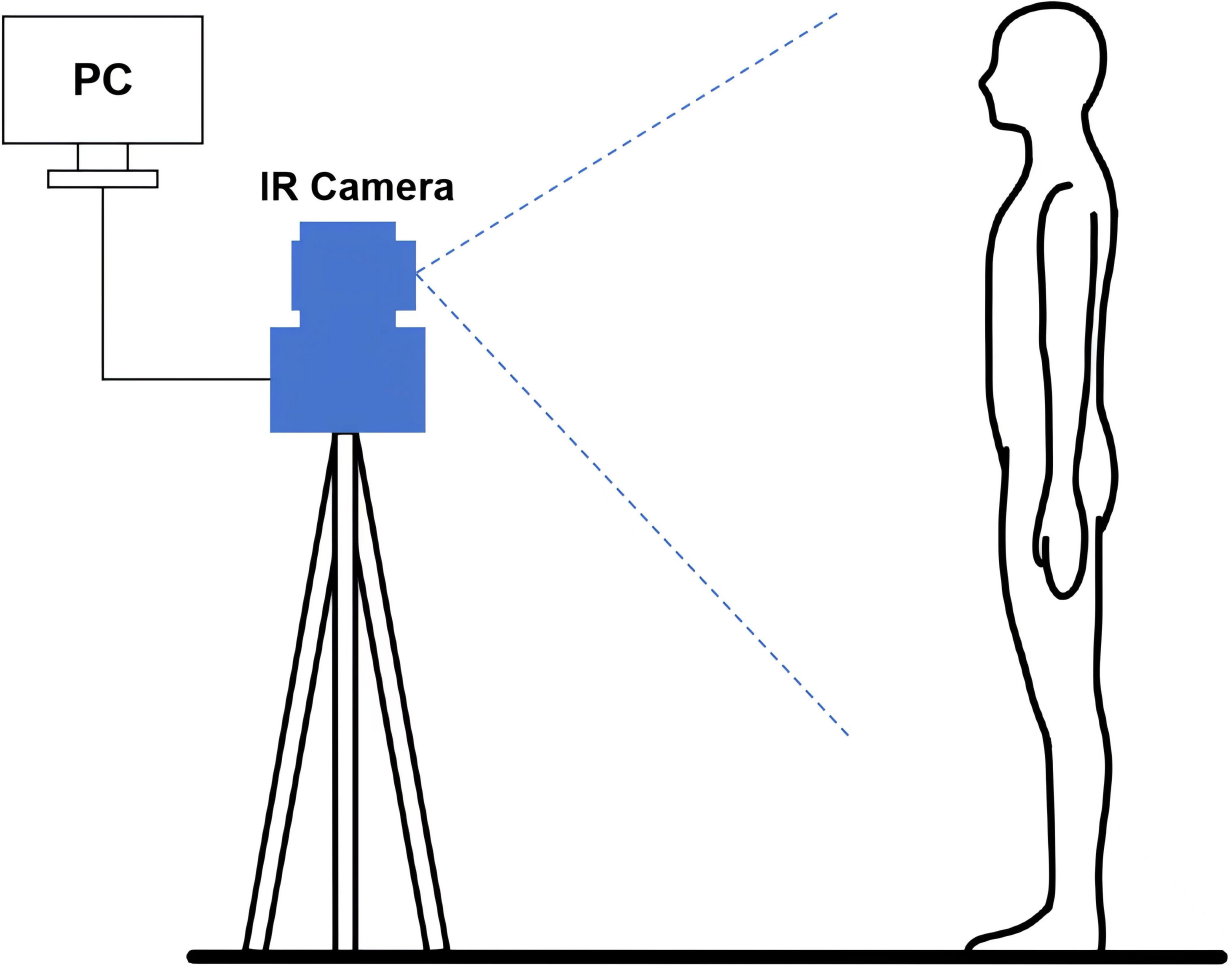
The flowchart for the literature search and screening process.

The acquisition of IRT data relies on high-performance equipment and appropriate collection methods, as illustrated in **Figure 2**. First, the selection of suitable IRT equipment should consider factors such as resolution, sensitivity, and sampling frequency. High-resolution infrared cameras can capture finer temperature variations, thereby enhancing data accuracy and analysis precision (36). The sensitivity of the equipment determines its ability to detect subtle temperature changes, which is crucial in mental health research. Because significant emotional fluctuations are often reflected in minor temperature variations. A higher sampling frequency facilitates the real-time recording of temperature changes, making it easier to capture rapid physiological responses.

With the advancement of technology, portable IRT devices and mobile applications have emerged, offering new conveniences for data collection. Portable devices allow data collection in natural environments, increasing the authenticity and diversity of the data. User-friendly interfaces of mobile applications simplify the data collection process, enabling non-professionals to perform basic data recording and analysis. Many portable IRT devices and mobile applications support real-time monitoring and analysis, providing immediate feedback to users, which is significant for instant mental health assessment and intervention.

IRT can collect temperature data in real time, typically equipped with high-frame-rate cameras capable of capturing 30 frames or more per second. During the experimental process, standardized procedures should be followed: ensuring the standardization of data collection, including fixed shooting distances, angles, and environmental temperature conditions, to minimize external interference and ensure data consistency and comparability. Additionally, it is usually necessary to mark and segment specific facial and body areas during the collection process to facilitate subsequent local temperature analysis. For example, commonly focused facial areas include the forehead, eye region, nose tip, and cheeks, as temperature changes in these areas are closely related to psychological states (37).

#### 3.3.2 Related technologies and data analysis methods

IRT is an important tool in mental health research due to its non-invasive, real-time, and highly sensitive characteristics. By monitoring temperature changes in different regions of the body, IRT provides physiological information related to an individual’s psychological state. The thermal distribution patterns of the face, limbs, and chest-abdominal areas serve as indicators for identifying psychological states. Temperature variations in these regions can be used to assess emotions, stress, and cognitive load. The following are common infrared data analysis methods.

The first step is analyzing thermal image. Thermal image analysis identifies physiological features of emotional and stress states by comparing facial temperature distribution patterns under different conditions. Image processing techniques such as edge detection and feature extraction are commonly used to identify temperature changes in key areas.

The second step is analyzing time series. Analyzing time series data of temperature changes reveals dynamic characteristics of psychological responses. Time series analysis methods such as Autoregressive Models (AR), Moving Average (MA), and Dynamic Time Warping (DTW) are widely applied to study trends in physiological signals.

The last step is integrating machine learning. Machine learning algorithms like Support Vector Machines (SVM), Random Forests (RF), and Deep Learning (DL) are used to analyze complex temperature patterns. These algorithms can handle high-dimensional data and extract meaningful patterns for emotion recognition and psychological state assessment. Multimodal Fusion: Combining IRT data with other physiological signals (e.g., Heart Rate Variability (HRV), GSR) for multimodal analysis can enhance the accuracy of mental health assessments. Multimodal fusion methods synthesize information from different data sources, providing a more comprehensive mental health evaluation.

The application of this technology relies not only on high-performance infrared imaging equipment and data analysis methods, but also on specific mental health application scenarios. As research progresses, IRT shows diverse potential applications in the mental health field for children and adolescents, especially in studies related to emotional disorders, ASD, PTSD, stress, and cognitive load. The following sections will explore the application of IRT technology in these specific mental health areas, analyzing its contributions to psychological state assessment and its future development prospects.

### 3.4 Application of IRT in emotional disorders assessment

Studies 1 to 5 in **Table 1** provide insights into the use of IRT for assessing anxiety symptoms. These studies illustrate how temperature changes in specific facial regions can be used to assess the severity of anxiety disorders. Three of these studies use thermal changes in facial regions as physiological indicators of anxiety levels and investigate the relationship between temperature variations in specific areas and anxiety severity (6, 7, 10).

Study 3 finds that thermal image analysis reveals anxiety-induced redirection of blood flow to the periorbital muscles (8). Study 5 uses IRT to examine correlations between thermal measurements of the nose and ears and metacognitive states during anxiety (9). IRT has been increasingly explored in recent years not only for the assessment of anxiety disorders but also in the study of depression disorders. Study 6 investigates the association between major depressive disorder (MDD) and corneal temperature (12). Study 7 uses IRT to identify five emotional states in children aged 7 to 11 years based on temperature information (11).

These studies suggest that IRT can serve as an objective tool for diagnosing anxiety and depression by detecting changes in thermal responses to emotional stimuli, demonstrating its potential utility.

### 3.5 Application of IRT in autism spectrum disorder assessment

According to Study 8 to 13 in the table, we can find that research on the application of IRT in ASD mainly focuses on two aspects: improving accuracy and building model datasets. In the context of leveraging multimodal physiological signals to improve both the accuracy and reliability of ASD detection and classification, two studies combined EEG data and facial thermography to classify ASD patients and neurotypical individuals (13, 18). Regarding the construction and provision of datasets using IRT for ASD detection, which facilitates the development and validation of deep learning models, four studies trained datasets based on thermal imaging of ASD children, all involving facial images. Emotion state model classifiers were proposed to distinguish between ASD and neurotypical children, with accuracy rates exceeding 90% (14–17).

### 3.6 Application of IRT in post-traumatic stress disorder assessment

According to Study 14 to 16 in the table, we highlight research on the application of IRT in PTSD, focusing on two key areas: enhancing early detection and identifying physiological biomarkers. In the context of improving early screening and prevention of PTSD, one study demonstrated that IRT could measure facial temperature changes due to blood flow variations by analyzing skin conductance responses (SCR) and facial thermal responses (38). Additionally, two studies combined IRT with SCR to assess fear regulation, aiding in the detection of minor PTSD symptoms [39, 40 (4)].

Regarding the identification of physiological biomarkers in severe PTSD cases, two studies monitored hemodynamic responses in the prefrontal cortex to capture specific changes associated with PTSD (19, 41). These studies found a strong correlation between IRT data and PTSD symptom assessments, allowing for precise evaluation of symptoms such as avoidance and hypervigilance. Furthermore, two studies integrated IRT with functional magnetic resonance imaging (fMRI) and near-infrared spectroscopy (NIRS) to provide a more comprehensive analysis of psychophysiological indicators (42).

IRT’s non-invasive nature offers significant potential for PTSD assessment. Future advancements in technology are expected to enhance IRT’s ability to detect minor temperature changes, supporting the development of personalized treatment plans. However, further research is needed to improve the accuracy of IRT, address ethical concerns, and standardize methods to ensure its broader application in PTSD assessment.

### 3.7 Application of IRT in stress assessment

Studies 17 through 21, as shown in the table, utilize thermal imaging to monitor changes in physiological indicators to assess stress levels. Research indicates that stress is associated with increased blood flow in the blood vessels of the forehead. These studies employ thermal imaging to observe emotional states. Other biological signals, including EEG, EMG, and pupil diameter, also exhibit changes under stress. Studies provide a comprehensive review of reliable biological signal indicators for detecting stress (43). High-resolution infrared imaging captures thermal patterns that are subsequently correlated with stress markers (21). The sympathetic nervous system triggers sweating in response to stress, and thermal imaging has been employed to quantify stress by measuring sweating responses in the nasal region (22). This method is effective for assessing stress, including in children (23). Additionally, the ‘accident paradigm’ used in conjunction with thermal infrared imaging helps observe facial temperature fluctuations in children, advancing research on stress in this demographic.

Some studies have used infrared machine vision (IRMV) technology and applied deep learning methods (44). One notable development is a deep learning model that uses thermal imaging to monitor nasal temperatures and breathing patterns for automatic stress recognition (24). Thermal imaging is increasingly being combined with various other technologies to enhance stress research. Furthermore, thermal imaging has been integrated with technologies such as photoplethysmography (PPG) to enable rapid stress detection. This integration highlights the potential for smartphone-based imaging systems to effectively monitor stress in real-time (25).

### 3.8 Application of IRT in cognitive load assessment

There are six studies focusing on the application of IRT in the context of cognitive load research. One study introduces the foundational theory of cognitive load, two studies investigate the mechanisms by which IRT measures cognitive load levels, and the remaining three studies examine the integration of IRT with cognitive load in other domains, including meditation, psoriasis, learning, and optimal training times. Details are provided in **Table 1**.

In the study of cognitive load measurement, growing attention is being given to physiological indicators such as heart rate, electromyography (EMG), blood volume, blood pressure, and body temperature (26, 27). IRT is increasingly utilized to measure cognitive load by detecting real-time changes in facial temperature. As IRT technology advances, its applications have broadened to encompass various types of cognitive load, including intrinsic, extraneous, and germane cognitive load (29–31).

## 4 Discussion

### 4.1 Principal results and comparison with prior work

In summary, IRT has been shown to be a reliable method for pervasive and automatic monitoring of psychophysiological activity. It provides a powerful tool for computational physiology. The reliability and validity of this technique can be demonstrated through evidence from multiple experiments described blow.

#### 4.1.1 Emotional disorders

Emotional disorders are psychological conditions characterized by disrupted emotional regulation, affecting an individual’s emotional expression, experience, and daily functioning. These disorders include anxiety disorders, depressive disorders, bipolar disorder, and other mood and affective disorders. Emotional disorders may arise from a variety of factors, including genetic, biological, psychosocial, and environmental influences. These disorders are especially prevalent among children and adolescents, potentially impairing academic performance, social interactions, and overall quality of life, thereby significantly impacting mental health (45). Therefore, early intervention is crucial to mitigate the long-term developmental effects of emotional disorders in children and adolescents by identifying, assessing, and addressing these conditions as early as possible (46).

**Table 1** above shows the relevant studies on the application of IRT in the assessment of emotional disorders. All studies use thermal variations in specific facial skin areas as primary measures to investigate their relationship with emotional disorders. Anxiety, depression, and other affective states associated with mood disorders influence vasodilation and vasoconstriction through the autonomic nervous system. This influence leads to a redistribution of blood flow within vessels, which can result in either an increase or decrease in skin temperature (47). Due to the thinness of facial skin, changes in blood flow in superficial vessels are more detectable, resulting in significant variations in skin temperature. By measuring facial skin temperature and analyzing its spatial and temporal distribution, these studies provide a biological basis for a deeper understanding of emotional disorders, particularly in younger populations.

These studies suggest that IRT technology holds considerable potential for monitoring anxiety and depression states and outline a common methodology for measuring and analyzing changes in facial temperature. The typical approach involves segmenting thermal images from an infrared thermograph 363 to define ROIs on the face, including the forehead, nasal tip, cheeks, mouth, chin, periorbital area, and 364 nasolabial folds. For each thermal frame, temperature averages, variances, and medians are calculated for 365 these ROIs. Linear discriminant analysis is then used as a classifier to analyze the data.

The studies indicate that anxiety is associated with increased temperatures in the temporal and frontal regions as well as at the tip of the nose, while depression is linked to a higher core body temperature, elevated temperatures around the right eye, and reduced efficiency in temperature regulation. Both anxiety and depression contribute to higher temperatures in the periorbital region. These findings highlight the effectiveness of IRT technology in reflecting emotional fluctuations, suggesting that temperature changes in specific facial regions can serve as biological markers for assessing the severity of anxiety disorders.

While IRT has been extensively used to analyze and identify emotional disorders in adults, its application in children and adolescents remains under-researched (11). Traditional methods for diagnosing emotional disorders include EEG, heart rate (HR), blood pressure (systolic and diastolic) and skin conductance response (SCR). However, these methods are invasive and require prolonged measurement, which may cause discomfort, especially in active children. Moreover, the large volume of data generated results in significant computational burdens. In contrast, the non-invasive and non-contact nature of IRT technology addresses the difficulties associated with skin sensors in children, offering significant convenience for screening and evaluating emotional disorders (47).

#### 4.1.2 ASD

Autism Spectrum Disorder is a complex neurodevelopmental disorder that generally appears early in childhood and continues throughout life. The disorder is characterised by core features in two areas—social communication and restricted, repetitive sensory–motor behaviours [48 (8)]. These challenges stem from the different ways individuals with ASD perceive and process information compared to those without the disorder. As a result, individuals with ASD often face significant challenges in social interaction and struggle with emotional expression. Additionally, individuals with ASD frequently experience sensory hypersensitivity or hyposensitivity. These sensory abnormalities can further impair their behavior and communication skills.

IRT’s ability to detect thermoregulatory abnormalities in individuals with ASD is particularly valuable for its role in ASD assessment. Research shows a correlation between facial temperature changes and the emotional and social cognitive states of children with ASD, suggesting that temperature fluctuations, like those caused by fever, may temporarily improve social and communication skills. These thermoregulatory variations are consistent across multiple sessions and have been associated with improvements in core ASD symptoms and brain connectivity, highlighting IRT’s potential as a valuable tool for identifying ASD-related biomarkers.

In addition to its diagnostic capabilities, IRT provides valuable insights into the physiological processes underlying ASD. It reveals aspects of sensory processing and emotional regulation that are often challenging to assess using traditional methods. By connecting physiological markers with behavioral symptoms, IRT improves the accuracy of ASD diagnosis and introduces new avenues for targeted intervention. Combining IRT with other physiological data, such as EEG, and applying advanced machine learning models, like CNNs, has significantly advanced the field of ASD research (13, 18). These advancements not only enhance detection rates but also facilitate the creation of personalized therapies tailored to each individual’s thermal profile, addressing their unique needs.

Applying deep learning models to IRT data highlights the significant impact of technology on improving ASD management strategies (14, 15). The creation of extensive datasets underscores IRT’s scalability and its applicability in clinical environments.

In summary, IRT’s role in ASD assessment extends beyond mere diagnostic capabilities. By combining physiological insights with advanced computational tools, IRT holds the potential to revolutionize both the diagnosis and treatment of ASD, leading to more effective and individualized interventions.

#### 4.1.3 PTSD

Post-Traumatic Stress Disorder is a complex anxiety disorder usually triggered by traumatic events. These events include accidents, physical injuries, sexual assault, crime, war, torture, or natural disasters like wildfires or floods. PTSD symptoms include re-experiencing, avoidance, and hyperarousal. Since facial temperature changes are linked to emotional experiences and psychological states, IRT can assess PTSD in real-time and non-invasively.

IRT holds significant potential in assessing PTSD, demonstrating its ability to study the emotional responses, stress reactions, and psychological states of PTSD patients. Many studies have showcased the potential of IRT in emotion detection, using an intelligent thermal system based on thermographic images for detecting human emotions. Cho et al. demonstrated that IRT could automatically recognize stress responses in an unconstrained environment using low-cost thermal imaging technology. Other studies tend to use higher-resolution cameras and advanced image processing algorithms to more precisely detect small temperature changes in PTSD patients, thereby more accurately reflecting their psychophysiological state. The advantage of IRT is that it can monitor physiological parameters in natural environments without interference, providing more comprehensive diagnostic information than traditional methods. As technology advances, improvements in IRT resolution and image processing algorithms are expected to further enhance its ability to detect subtle temperature changes and reflect psychophysiological states. The development trend of IRT technology indicates that it will play an increasingly important role in future clinical applications, such as the formulation of personalized treatment plans. In the future, IRT may develop personalized stress response models in PTSD research, which will help better understand individual differences and create more personalized treatment plans in clinical applications.

Although IRT has broad prospects in PTSD research, it still faces challenges in technology, ethics, and application environments. Future research needs to improve technical accuracy and data interpretation while addressing related ethical issues to promote the widespread application of IRT technology in the PTSD field. The standardization of methods and validation of data may become a focus of future research. Despite these challenges, IRT continues to open new perspectives for PTSD research and treatment. Its application in PTSD research not only provides effective means for monitoring emotional and stress responses but also enables non-invasive real-time monitoring, with the potential to expand its application and improve its effectiveness in future studies on trauma recall triggers.

#### 4.1.4 Cognitive load

Cognitive load in cognitive psychology refers to the amount of working memory resources required for processing information. In simpler terms, it represents the mental and cognitive resources an individual needs to process tasks. The cognitive load varies based on task complexity and the individual’s cognitive capacity. Cognitive load is typically categorized into three types: Intrinsic Cognitive Load (ICL), determined by the inherent complexity of the learning material; Extraneous Cognitive Load (ECL), resulting from poorly designed instructional environments; and Germane Cognitive Load (GCL), associated with the construction and retrieval of mental schemas. Germane Cognitive Load involves the allocation of working memory resources to cognitive processes, including the storage and application of mental images (49). Since cognitive processes primarily occur within the brain and are not directly observable, capturing information about cognitive states presents significant challenges. Relying solely on user introspection often fails to yield an accurate and objective representation of cognitive processes. Research indicates that measuring physiological indicators such as body temperature, heart rate, and electromyography (EMG) can provide valuable insights into changes in cognitive load.

Research indicates that many cognitive load measurement methods involve invasive techniques, such as body-attached sensors or psychological assessment scales. Non-contact measurement methods help reduce participant discomfort and avoid the physiological and psychological interference associated with sensor attachments. IRT represents a departure from traditional subjective measurement methods, playing a crucial role in the non-invasive, objective, and real-time measurement of cognitive load. IRT represents a departure from traditional subjective measurement methods, playing a crucial role in the non-invasive, objective, and real-time measurement of cognitive load. For instance, researchers such as Or et al. (27) simulated both driving and real-world environments. They employed IRT to measure participants’ facial temperatures in real-time, comparing temperature data between experimental and control groups. This approach demonstrated the feasibility of using infrared thermography for psychological load measurement. Commercial thermal imaging cameras have also been utilized by researchers such as Abdelrahman et al. (26) to measure cognitive load levels. They utilized indicators such as breathing rate, nasal temperature, and heart rate to examine cognitive load during mental labor and fatigue in a digital manufacturing environment. They found that nasal temperature, as measured by infrared thermography, can effectively assess task demands and contribute to more efficient workflow design. Various experiments have confirmed the feasibility of using IRT for cognitive load measurement. Cognitive load measurement is no longer solely focused on cognitive load itself. As research progresses, cognitive load studies have expanded from a single dimension to multidisciplinary and cross-scenario research, integrating with fields such as meditation, psoriasis, learning, and optimal training times. For example, in assessing intrinsic cognitive load, Wang R et al. (29) combined infrared imaging with meditation, using facial temperature data to study meditation behavior. They found that an increase in chin temperature and a decrease in forehead temperature indicated a meditative state. Regarding extraneous cognitive load, Panasiti et al. (30) applied IRT to assess emotional self-regulation in psoriasis patients under varying cognitive load conditions. They explored cognitive strategies that are suitable for emotional healing.

For germane cognitive load, Kang et al. (31) utilized IRT to examine the relationship between cognitive load, learning levels, and optimal training times. They found that nasal temperature changes with new learning tasks, but these changes were not sustained, and extending learning time did not necessarily enhance learning efficiency. Cognitive load has emerged as a new indicator in psychological research across various fields. IRT enables the exploration of cognitive load effects in various scenarios, providing deeper insights into research questions within this field. Future research will likely see cognitive load increasingly integrated across multiple disciplines, serving as an indicator of psychological health.

#### 4.1.5 Stress

Stress helps humans cope with perceived or real threats and challenges. It plays a crucial role in task execution (50). IRT is widely used to assess stress responses in children and adolescents. With its remote and non-invasive characteristics, it can significantly reduce the impact of subjects’ subjective reactions on experiments, providing tremendous assistance in stress assessment. Research shows that facial temperature, especially around the eyes and nose, is closely related to the autonomic nervous system’s activity. This characteristic serves as the basis for using IRT to assess stress levels. Through IRT’s real-time monitoring of temperature, physiological signals become the medium that connects IRT with the human body’s stress levels. Many scholars link autonomous emotional changes with objective physiological indicators, enabling the assessment of subjects’ stress levels without being influenced by subjective thinking. This physiological measurement method provides a highly effective approach for stress assessment and feedback.

With technological advances, more researchers are focusing on the integration of thermal imaging technology and deep learning. By developing deep learning models, researchers can obtain more accurate results from thermal imaging temperature recognition, as well as a more rational and comprehensive temperature recognition system, thereby enabling a more precise assessment of stress levels. Furthermore, with the continuous development by researchers, an increasing number of technologies are being integrated with infrared thermography, contributing to the enhancement of stress assessment. These studies demonstrate that detecting temperature through infrared thermal imaging is an effective method to assess stress levels.

### 4.2 Summary

In conclusion, the previous discussion emphasizes the considerable potential of IRT technology in mental health diagnostics. IRT can detect psychophysiological changes linked to emotional disorders, such as somatic responses to anxiety and depression. It is also capable of real-time monitoring of PTSD and identifying stress triggers. Additionally, IRT captures facial temperature variations resulting from stress, cognitive load-induced fatigue, and attention decline, which demonstrates its sensitivity in detecting emotional changes. Furthermore, IRT offers valuable insights into physiological changes related to emotion regulation, social cognition, and sensory processing in ASD, thereby providing new opportunities for a more profound understanding of ASD.

Numerous studies have highlighted the capabilities of IRT in psychophysiology, particularly in detecting human thermal variations using advanced thermal imaging systems. Several studies have integrated IRT with other physiological measurement devices. For example, IRT can been combined with a GSR sensors with piezoelectric chest strips to monitor respiration and detect thermal changes alongside synchronous physiological responses, such as variations in skin resistance and respiratory rhythm. By integrating thermal imaging with other biometric techniques, these approaches first generate a comprehensive data set that improves the identification of psychological disorders and enhances result reliability. Additionally, the consistency between physiological and thermal imaging data supports the effectiveness and accuracy of IRT technology.

The effectiveness of IRT in mental health relies on advancements in thermal infrared imaging technology. Modern devices, with their high spatial and temporal resolution and enhanced thermal sensitivity, support the integration of IRT into automated systems (19). For instance, M. Melinda et al. (15)have developed intelligent systems for remote, automated monitoring of physiological activities, which facilitate real-time processing and classification of thermal infrared image data for psychophysiological applications. Beyond psychophysiology, the advantages and characteristics of IRT extend its application in developmental psychology, social psychology (21), and zoology (51). Given that IRT detects autonomic nervous system thermal signals, it is effective for monitoring autonomic regulation and suitable for long-term, remote, non-contact, and frequent assessments.

As research highlights, IRT offers a promising and effective technological approach for studying PTSD, cognitive load, and other areas due to its non-invasive and real-time monitoring features. While IRT has been widely used in adult mental health and psychiatric assessments, its application in pediatric populations is still limited (23). Traditional medical examinations often cause anxiety in younger patients, but IRT’s non-invasive nature enhances comfort and reduces psychological stress, facilitating a more comfortable examination experience. Furthermore, since children and adolescents are generally more active during monitoring, IRT’s non-contact approach accommodates their movements and avoids measurement errors associated with direct contact, thus improving overall measurement accuracy.

Currently, despite its numerous advantages, IRT remains primarily in the experimental phase within research settings and has not yet achieved widespread adoption in clinical diagnostics. Physicians generally prefer well-established methods, such as psychological assessment scales and biofeedback, due to their extensive validation and integration into clinical practice. The limited adoption of IRT may be attributed to its ongoing validation and lack of standardization. Additionally, the interpretation of IRT data may be complex, often requiring integration with other physiological and psychological data, which can increase the workload and complexity for clinical settings. These factors contribute to the relatively limited use of IRT in clinical environments compared to traditional methods. However, with ongoing technological advancements and increasing research efforts, IRT may see broader clinical application in the future.

Recent advancements in infrared technology and the miniaturization of infrared cameras have led to the development of more compact and portable thermal imaging devices. This progress has helped mitigate the issue of high costs exceeding hospital budgets. For example, companies such as FLIR (Wilsonville, Oregon, USA) have introduced portable thermal imaging devices that can be integrated with smartphones. These devices offer significant advantages for various medical applications, including psychological health monitoring. Their flexibility helps medical personnel reduce the cumbersome steps of traditional monitoring methods, enabling quicker monitoring and assessment, and optimizing resource allocation.

In summary, IRT demonstrates considerable potential in the field of mental health. IRT could improve the accuracy and reliability of detecting certain psychological issues and mental disorders by providing diverse data that supports comprehensive mental health assessments. While IRT remains experimental in clinical settings, its portability and non-invasive nature are progressively reducing barriers to its use, suggesting potential benefits for psychological evaluation, particularly in children and adolescents. With continued clinical validation and research, IRT is anticipated to become a valuable tool in mental health, potentially facilitating more precise diagnostic and treatment approaches.

## 5 Limitations

### 5.1 Limitation of the effectiveness of IRT technology in mental health assessment

The aforementioned studies on mental health assessment and diagnosis using IRT have shown affectiveness in monitoring temperatures variations across body regions. However, these studies have not progressed to being used for diagnostic purposes. This limitation is partly due to the lack of automated systems for identifying emotional disorders and partly because the accuracy of IRT can be affected by environmental temperature and humidity. The accuracy of IRT is highly dependent on the quality of thermal imaging equipment and environmental conditions, which can introduce variability in results. Moreover, the rapid review methodology adopted in this study, while efficient and practical for providing an overview of the literature, inherently carries certain methodological limitations. The search is restricted to English-language articles indexed in selected databases, which may have led to the exclusion of relevant studies published in other languages. Although inclusion and exclusion criteria are systematically applied, the screening process may still involve a degree of subjectivity, potentially affecting the consistency of study selection. Furthermore, this review may be subject to publication bias, as studies reporting statistically significant or positive results are more likely to be published and included in academic databases, while studies with null or negative findings may remain unpublished or less accessible. This could result in an overrepresentation of favorable outcomes and a less comprehensive view of the existing evidence. Finally, although this review aims to explore the application of IRT in pediatric mental health, only a limited number of included studies focused specifically on children and adolescents, which may restrict the generalizability of the findings to this population.

The different physiological and skin characteristics of the subjects also present another significant challenge in the application of IRT, affecting the accuracy of temperature measurements. This variability complicates cross-individual comparisons and analyses, making it difficult to establish standardized references and impacting the reliability of research and diagnostic outcomes. In pediatric-focused studies, facial temperature, especially in regions such as the nose, eyes, and forehead, often show significant changes during emotional tasks. For example, Ganesh et al. (14) use CNN-based models to classify autistic children with greater 96% precision based on thermal features. While these findings are promising, pediatric-specific data remains limited relative to adult-focused research, underscoring the need for more age-targeted investigations and population-specific validation.

Furthermore, IRT primarily infers emotional responses by measuring facial temperature changes. However, these temperature changes are not always directly related to psychologically induced physiological reactions and may be insufficient to independently identify the complex physiological and psychological reactions associated with the disorder. According to Glenn J. Tattersall (51), infrared thermal imaging may lack specificity for detecting emotional changes. Therefore, further research is necessary to establish the specificity and sensitivity of thermal imaging as a tool for detecting anxiety or depression.

### 5.2 Limitation of IRT as a stand-alone diagnostic tool

Additionally, IRT provides valuable insights into emotional and cognitive states. Although IRT technology can intuitively detect changes in physiological indicators and is unaffected by subjective factors of the tester, IRT itself does not have the ability to directly diagnose mental health conditions. Body temperature changes may reflect various physiological or psychological states, but it is difficult to infer specific emotions or symptoms from IRT data without corroboration from additional physiological indicators. Therefore, it should be used as a complementary tool rather than a standalone diagnostic method, given its limitations in capturing the full complexity of mental health conditions. The data from IRT should be used as supplementary evidence, integrated with psychological theories, behavioral observations, standardized assessment tools, and other multi-dimensional information for a thorough analysis. Although analysis of IRT data can be combined with multimodal data inputs such as EEG, this requires complex computational models, making it less amenable to widespread clinical use.

Besides, despite of the advantages offered by thermal IR imaging, it has to be taken into account that thermal signal development as a result of vascular change, perspiration, or muscular activity is rather slow with respect to other established techniques. Therefore, proper considerations should be taken when monitoring thermal signatures of psychophysiological activity.

### 5.3 Limitation of IRT technology in laboratory environments

According to the research reviewed, most of the studies have been conducted in laboratory or controlled environments. In these settings, the stimuli designed to simulate emotions such as anxiety are usually intense to elicit prominent and measurable biological responses. However, in real life conditions, real-life stimul i are usually complex and multifaceted, involving many aspects of human personality and multiple concurrent events due to the intricacies of everyday life.

## 6 Future work with artificial intelligence

The integration of IRT and Artificial Intelligence (AI) holds significant promise for advancing mental health care. It provides innovative approaches for early psychological screening, personalized mental health interventions, and advanced diagnostic tools. This integration is reflected in innovative applications, including data processing, precision medicine, real-time monitoring and feedback, and multimodal data fusion.

### 6.1 Efficient data processing and analysis

As mental health issues become increasingly prevalent, researchers are increasingly focusing on efficient data processing and analysis for early disease screening and detection. IRT technology offers high temporal resolution, enabling the capture of subtle fluctuations in body temperature. This capability facilitates the application of AI models in mental health by providing rich, time-sensitive data for analysis. IRT data can be processed and analyzed using machine learning and deep learning algorithms, enabling the handling of extensive datasets (52). As a result, AI can extract relevant disease characteristics from IRT data, facilitating early detection of mental health issues (53). Building proprietary large-scale models for mental health screening using IRT technology is both necessary and feasible. IRT technology offers advantages such as non-invasiveness, convenience, and dynamic monitoring in the field of mental health, making it suitable for large-scale population screening studies. By accumulating extensive data, AI large-scale models can provide more accurate assessments for mental health evaluation, revealing potential mental health risks.

### 6.2 Precision medicine and intervention

The combination of IRT and AI can be utilized to develop precise mental health intervention strategies (52). AI models can analyze physiological data collected by IRT to identify unique psychological states and patterns of emotional change in individuals. This personalized analysis helps clinicians customize the most effective treatment plans for each patient, thereby enhancing the effectiveness of mental health interventions.

### 6.3 Real-time monitoring and feedback

Real-time monitoring and feedback are critical in the clinical assessment, intervention, and follow-up processes of mental health care. IRT possesses the capability for real-time monitoring, enabling the capture of physiological changes in patients without disturbance (2). When integrated with AI technology, this monitoring capability enables more objective and data-driven assessments of patient outcomes. Real-time analysis of temperature changes using AI algorithms allows for immediate feedback, enabling clinicians to adjust treatment strategies to optimize outcomes.

### 6.4 Multimodal data fusion and intelligent decision making

Multimodal data fusion represents another crucial direction in the integration of IRT and AI. In mental health assessment, relying solely on a single data source may lead to inaccurate and incomplete outcomes. By combining IRT data with other psychophysiological data sources such as facial expression recognition, heart rate variability, audio, and video, a more comprehensive analysis can be achieved (54). This multimodal data fusion provides a more objective, authentic, and accurate auxiliary decision-making system. Integrating IRT data with other physiological signals through intelligent decision-making algorithms significantly enhances the accuracy of mental health assessment and reduces the impact of subjective judgment. This approach not only supports more accurate auxiliary diagnosis but also informs more effective clinical decision-making, ultimately leading to better patient outcomes.

**Figure 3:**
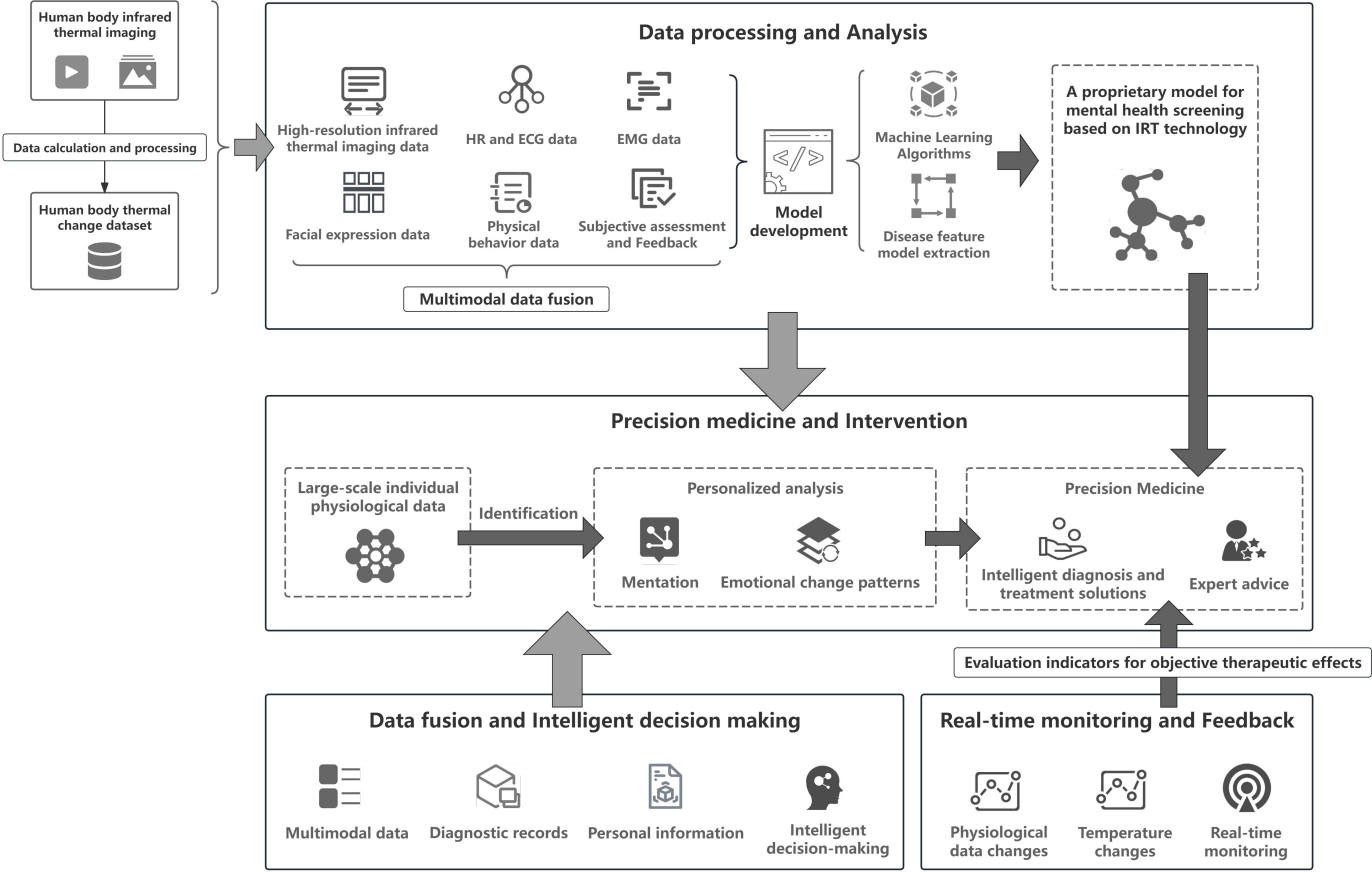
Application of IRT combined with AI in the field of mental health in the future.

The schematic diagram of the future application of IRT combined with AI in the field of mental health is shown in **Figure 3**.

## 7 Conclusions

This paper provides a comprehensive review of the application of IRT in the study of mental health among children and adolescents. As a non-invasive and real-time monitoring tool, IRT offers a new perspective in the field of mental health. Through studies on emotional disorders, ASD, PTSD, stress, and cognitive load, the unique advantages of IRT in detecting and assessing psychological states are demonstrated. Despite its broad potential applications, IRT faces several challenges in the mental health domain. For instance, the complexity of IRT data analysis and the integration with other physiological and psychological indicators require further exploration.

Looking forward, the integration of IRT with AI is a promising development. With the support of AI technology, IRT can faciliate more intelligent and personalized mental health assessments. This integration not only improves the accuracy of assessments but also fosters personalized mental health interventions. Future research can focus on multimodal data fusion, combining physiological, behavioral, and environmental data to provide more comprehensive mental health assessments. Additionally, IRT holds significant potential for application in telemedicine, offering sustainable mental health service solutions for resource-limited areas.

In summary, IRT has a broad application prospect in mental health research, with significant research value and practical significance. As technology continues to advance and interdisciplinary collaboration strengthens, IRT is expected to provide more innovative and effective tools and methods for mental health assessment and intervention in the future.

## Data Availability

The original contributions presented in the study are included in the article/supplementary material. Further inquiries can be directed to the corresponding author.
